# Analysis of exacerbating factors of pediatric asthma before and after the COVID-19 pandemic^☆^

**DOI:** 10.1016/j.waojou.2024.100961

**Published:** 2024-09-09

**Authors:** Youn Kyoung Won, Sung-Il Cho, Eun Hee Chung

**Affiliations:** a Department of Pediatrics, Pocheon Woori Hospital, Pocheon-si, Gyeonggi-do, South Korea; b Department of Public Health Science, Graduate School of Public Health, Seoul National University, Seoul, South Korea; c Department of Pediatrics, Chungnam National University School of Medicine, Daejeon, South Korea

**Keywords:** Asthma, COVID-19, Children, Adolescents, Emergency department, Respiratory virus

## Abstract

**Purpose:**

The incidence of the existing respiratory virus and air pollutants had disappeared or decreased due to social distancing during the coronavirus disease 2019 (COVID-19) pandemic. Therefore, there was no increase in asthma exacerbations in 2020. This study aimed to analyze the emergency department (ED) visits of children and adolescent patients with asthma before and after the COVID-19 outbreak and examine the effects of respiratory virus infection and air pollutants.

**Methods:**

This study included pediatric and adolescent patients with asthma aged 2–18 years who visited 419 EDs nationwide during February to December in 2018, 2019, and 2020. The patients who were diagnosed with asthma, ie, J45 or J46 (International Classification of Diseases, 10th revision) in the ED medical history, diagnosis history at discharge, and diagnosis at discharge after hospitalization through the ED were included using the National Emergency Department Information System. Data were analyzed by dividing the period as follows: pre-COVID-19 (from February to December 2018 and 2019) and COVID-19 pandemic (from February to December 2020).

**Results:**

The monthly average of 673 visiting patients (95% confidence interval [CI], 474–872) during the pre-COVID-19 period decreased to 176 (95% CI, 113–239) during the COVID-19 pandemic, which is a 73.8% decrease (p < 0.001).

In the pre-COVID-19 period, peaks were observed in spring and autumn. Meanwhile, during the COVID-19 pandemic, a peak was observed only during autumn. During the COVID-19 pandemic, no relationship was found between the rhinovirus infection and asthma exacerbations (p < 0.001).

**Conclusions:**

Respiratory virus infections are strongly associated with asthma exacerbations in children and adolescents. In this study, air pollution is not a major factor for ER visits due to asthma exacerbations. Even though the prevalence of respiratory viruses is decreasing, ED visits due to worsening asthma are trending in the fall. This phenomenon may indicate that asthma has worsened due to other causes such as pollen or fluctuations in temperature and air pressure.

## Introduction

Asthma is a chronic disease characterized by recurrent and reversible airway obstruction, airway hyperresponsiveness to various stimuli, and chronic airway inflammation,^1^ where respiratory symptoms exacerbate and improve repeatedly. Factors known to be involved in the manifestation and exacerbation of asthma symptoms include respiratory infections, allergens, air pollution, changes in temperature, and atmospheric pressure. Considering respiratory infections, which are known to account for the largest share of the causes of asthma exacerbations in children and adolescents, Johnston et al^2^ performed a respiratory virus test in children aged 9–11 years who had asthma exacerbations, and detected respiratory viruses in 80–85% of them, especially during spring and autumn. Furthermore, the pattern of detection of rhinovirus coincided with the increase in hospitalization due to asthma in spring and autumn. In South Korea, the peak of patients with asthma exacerbations was shown in September and October of each year, but there was a surge in the number of patients who visited the emergency department (ED) due to asthma exacerbations from October to December 2009, the period of the novel influenza virus epidemic.^3^ A previous study on 10 European cities regarding air pollution,^4^ which is another cause of asthma exacerbations, found that exposure to air pollutants related to road traffic caused asthma exacerbations in 15% of pediatric patients.

During the coronavirus disease 2019 (COVID-19) pandemic, respiratory viral infections and air pollution, the main causes of asthma exacerbations, decreased. Since the beginning of the COVID-19 pandemic, many countries have implemented non-pharmacological interventions to prevent the spread of the virus. After the first confirmed case of COVID-19 in South Korea on January 20, 2020, even before the government officially enforced social distancing, people voluntarily wore masks and refrained from engaging in outside activities.^5^ On March 22, 2020, the South Korean government established the Central Disaster and Safety Countermeasures Headquarters for COVID-19, which was composed of experts from various institutions and implemented nationwide social distancing. Basic rules for distancing in daily life were announced, and people with symptoms were tested and isolated. In addition, social distancing was applied in stages to limit the number of people in gatherings according to the daily number of confirmed cases in the community, while public institutions and companies recommended telecommuting.^6^ Elementary, middle, and high schools postponed the start of school in March 2020 and continued to remain closed until May 20, 2020. While schools gradually reopened by June, strict social distancing measures were still enforced, with limited group activities.^5^ These nationwide interventions reduced respiratory virus infections^7^ and air pollution levels as people were restricted from social activities.^8^ This suggests the effects of respiratory viral infection and air pollution on asthma exacerbations in children and adolescents.

This study aimed to investigate the effects of respiratory viral infection and air pollution reduction during the COVID-19 pandemic on asthma exacerbations in children and adolescents. The number of children and adolescent patients with asthma who visited the ED nationwide from February to December 2020, when social distancing was implemented with the onset of the COVID-19 pandemic, was compared with that before the COVID-19 pandemic, from February to December 2018 and from February to December 2019.

## Materials and methods

This study included pediatric and adolescent patients with asthma aged 2 to 18 years who visited 403 EDs nationwide from February to December in 2018, 2019, and 2020. A total of 16,735 patients diagnosed with asthma according to J45 and J46 (International Classification of Diseases, 10th revision) were included based on the ED treatment records and diagnosis records upon discharge at ED and after hospitalization through the ED. The number of pediatric and adolescent patients who visited the ED due to asthma exacerbations was confirmed by requesting data from the National Emergency Department Information System (NEDIS).

The number of respiratory viruses presented as an independent variable in this study was collected from January 2018 to December 2020 using the Pathogens & Vector Surveillance Weekly Report (PVSWR) website,^9^ one of the publications by the Korea Disease Control and Prevention Agency (KDCA).

The Korea Influenza & Respiratory Viruses Surveillance System (KINRESS) operates by receiving notifications from sentinel surveillance agencies designated in accordance with the Infectious Disease Control and Prevention Act, and consists of “influenza-like illness surveillance” implemented since September 2000 and “inpatient surveillance for influenza and acute respiratory infections” for hospital-level healthcare institutions implemented since 2011.^10^ However, since selection bias may occur when detecting viruses in patients with high severity, such as inpatients, the test results from outpatients, “influenza-like illness surveillance” reports, were used. “Influenza-like illness surveillance” reports the laboratory test results on samples from patients suspected of having acute respiratory viral infection sent from primary healthcare institutions (52 pediatric, internal medicine, and family medicine departments) located in 18 cities/provinces in Korea.^9^

KINRESS is responsible for the characterization of causative pathogens and identification of epidemic patterns of 7 types of influenza and respiratory viruses found across the country. Participating healthcare institutions from throughout Korea provide respiratory samples (oropharyngeal and nasopharyngeal smears) from patients with influenza-like illness and patients with acute respiratory infection that are collected every week, while 18 city/provincial research institutes of public health and environment perform gene detection test via real-time reverse transcription-polymerase chain reaction (RT-PCR) on 7 types of influenza and respiratory viruses (respiratory syncytial virus, adenovirus, rhinovirus, parainfluenza virus, metapneumovirus, coronavirus, and bocavirus) in the collected samples. KDCA pools and analyzes the gene detection test results and publishes the findings on a weekly basis in the infectious disease portal (Sentinel Surveillance Weekly Report and PVSWR) found on the KDCA website.^11^

The air pollution index, presented as another independent variable, was obtained from January 2018 to December 2020 using the Air Korea website^12^ provided by the Korean Ministry of Environment and the Korea Environment Corporation (K-eco). The air pollution index was calculated as the monthly average concentration of air pollutants (sulfur dioxide [SO_2_], nitrogen dioxide [NO_2_], ozone [O_3_], carbon monoxide [CO], and particulate matter [PM2.5 and PM10]) in 7 large cities of South Korea (Seoul, Incheon, Daejeon, Daegu, Gwangju, Busan, and Ulsan) with a population of 1 million or more.

As the study was conducted using publicly available data, there was no requirement for informed consent. The study protocol was approved by the Ethics Committee of Ethics Committee of the Chungnam National University School of Medicine (IRB: CNUH-2021-06-056).

### Statistical analysis

The number of patients who visited the ED due to asthma exacerbations and the number of patients who were hospitalized after visiting the ED were confirmed using descriptive statistics, and the characteristics of the distribution by year were confirmed after categorizing by sex and age. The average monthly ED visits before and during the COVID-19 pandemic were compared using the independent samples *t*-test. During the COVID-19 pandemic, whether the monthly rate of patients who visited the ED with asthma exacerbations and the monthly detection rate of rhinovirus coincided was examined using the Chi-squared test. Analysis was performed using R statistical software (version 4.0.3; R Foundation for Statistical Computing, Vienna, Austria).

## Results

### Changes in respiratory virus detection

**Fig. 1:**
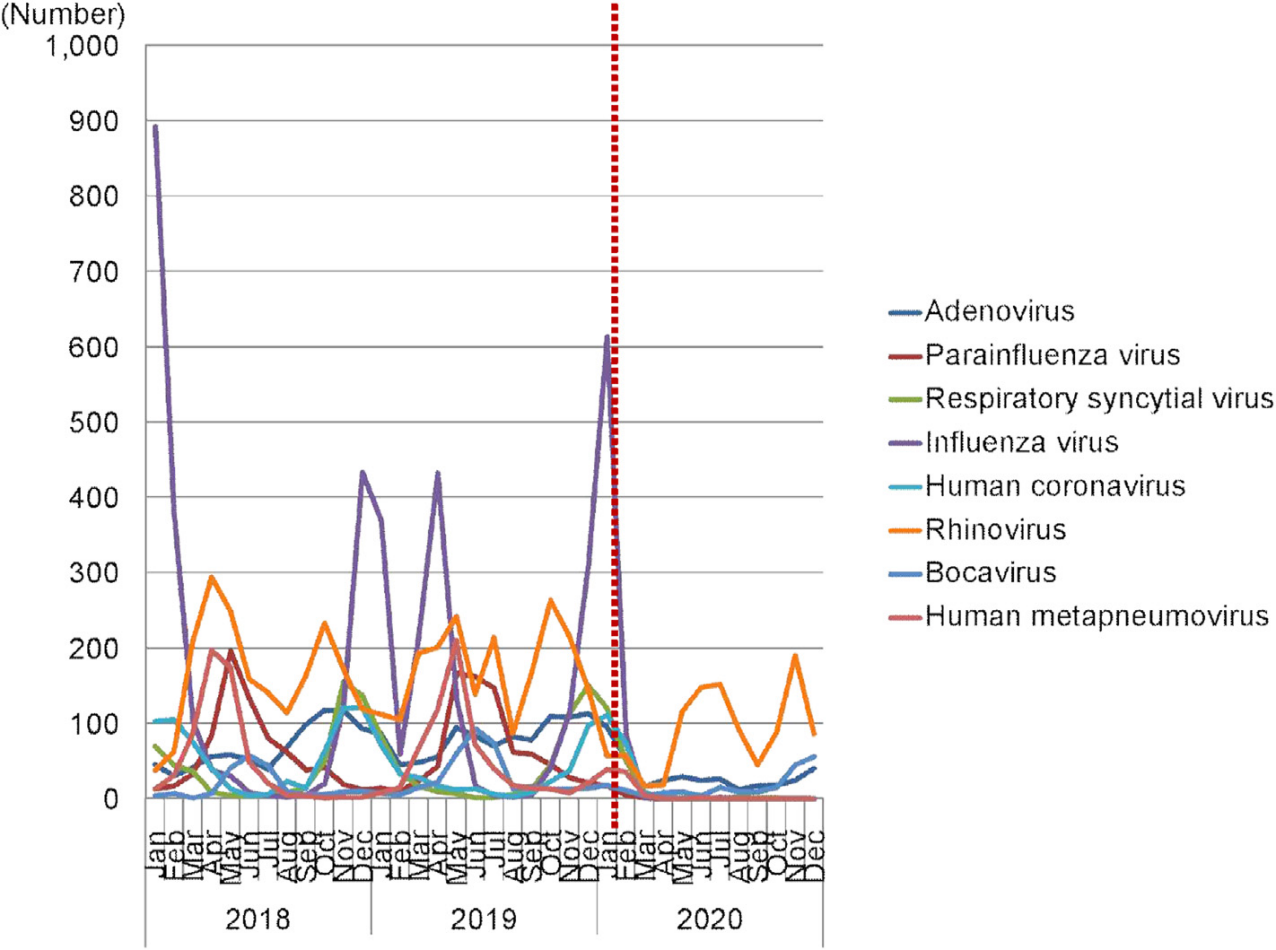
Changes in the number of respiratory viruses detected from 2018 to 2020. The dotted line represents the start of the coronavirus disease pandemic and social distancing in South Korea

The total number of samples in 2018, the period before COVID-19, was 11,965, where viruses were detected in 7,535 (63.0%) of the samples. In 2019, the total number of samples was 12,151, with viruses detected in 7,311 (60.2%) of the samples. During the COVID-19 pandemic in 2020, the total number of samples was 5819, of which viruses were detected in 2,832 (48.7%) of the samples. During the COVID-19 pandemic in 2020, the number of respiratory viruses showed a sharp decrease compared to the previous 2 years. In terms of the number of viruses detected during the COVID-19 pandemic in 2020, the detection rate of rhinovirus was the highest (see Fig. 1).

### Changes in air pollution

**Fig. 2:**
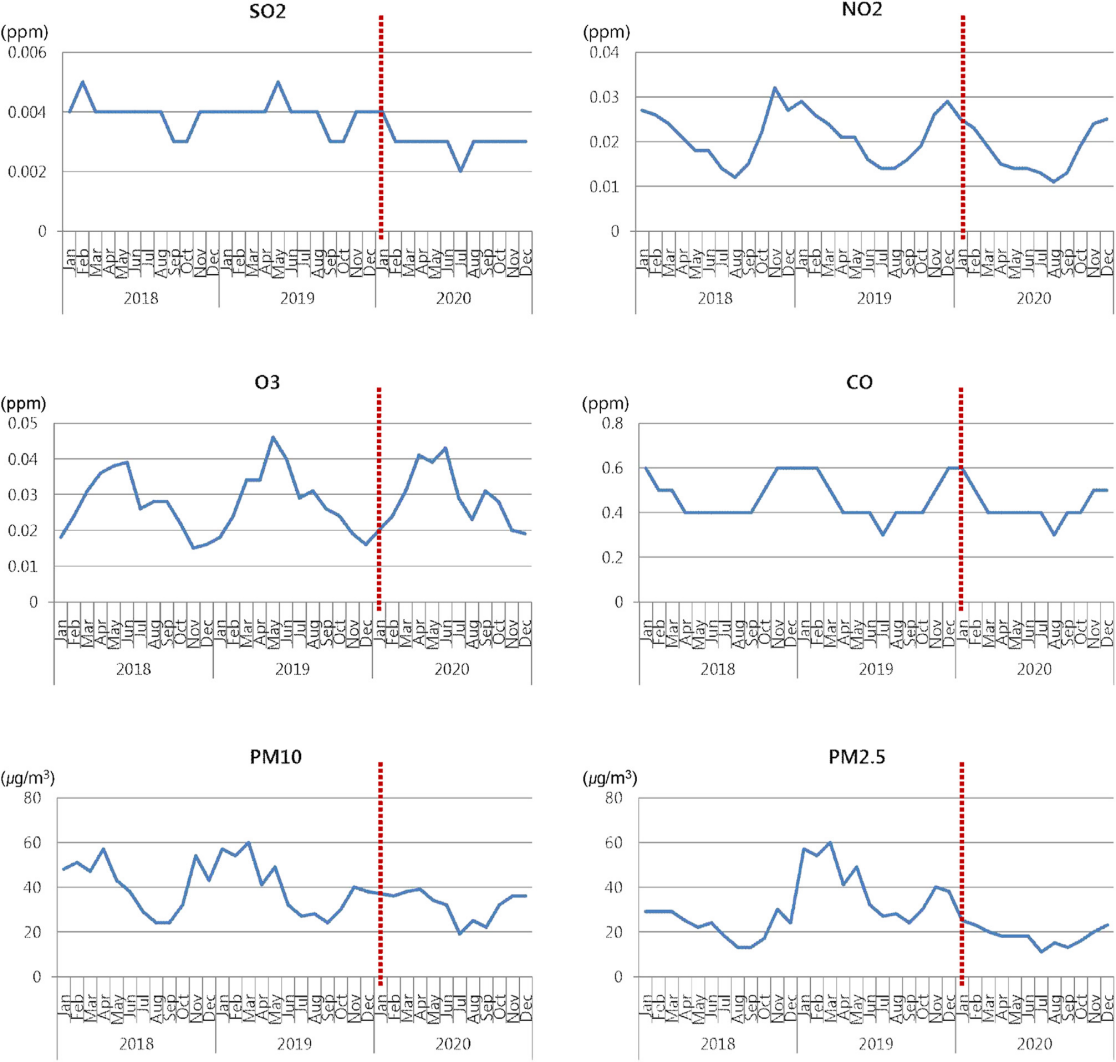
Changes in the air pollutant levels from 2018 to 2020. The dotted line represents the start of the coronavirus disease pandemic and social distancing in South Korea. PM, particulate matter.

The air pollution data showed that the SO_2_ level was 0.004 ppm per year before COVID-19 and decreased to 0.003 ppm during the COVID-19 pandemic (P < 0.001). NO_2_, O_3_, and CO showed the same concentration levels during the COVID-19 pandemic as that before the COVID-19 pandemic. PM10 decreased during the COVID-19 pandemic (average monthly concentration, 40 ppm (pre-COVID-19) and 32 ppm (COVID-19 pandemic), P = 0.03). PM 2.5 was higher in 2019 than in 2018 and 2020 (monthly average concentration, 22.8 μg/m^3^ (2018), 40.0 μg/m^3^ (2019), and 18.3 μg/m^3^ (2020); P < 0.001), and during the COVID-19 pandemic in 2020, it was at a similar level to that in 2018. Of the air pollution indicators, only SO_2_ and PM10 decreased during the COVID-19 pandemic (Fig. 2).

### Changes in ED visit rates in pediatric and adolescent asthma patients

During the study period, 16,735 patients aged 2 to 18 years old who visited 403 EDs nationwide were diagnosed with asthma. Before the COVID-19 pandemic, 7,822 patients visited the hospital from February to December 2018 and 6,981 patients from February to December 2019. During the COVID-19 pandemic, from February to December 2020, the number of patients decreased sharply compared to the previous 2 years, to 1,932. The number of patients hospitalized after ED treatment was 3,070 from February to December 2018 and 2,575 from February to December 2019. From February to December 2020, during the COVID-19 pandemic, there were 639 cases, showing a decrease from the previous 2 years (Table 1).

**Table 1:** Demographic characteristics of patients with asthma who visited the emergency department (ED) from February to December in 2018, 2019, and 2020.

Characteristic	Feb. to Dec. 2018	Feb. to Dec. 2019	Feb. to Dec. 2020	*P*-value
	N = 7,822	N = 6,981	N = 1,932	
Sex, n (%)				0.005
Male	4,952 (63.31%)	4,426 (63.40%)	1,151 (59.58%)	
Female	2,870 (36.69%)	2,555 (36.60%)	781 (40.42%)	
Age group, n (%)				<0.001
2−6 years	4,086 (52.24%)	3,276 (46.93%)	773 (40.01%)	
7−12 years	2,359 (30.16%)	2,341 (33.53%)	665 (34.42%)	
13−18 years	1,377 (17.60%)	1,364 (19.54%)	494 (25.57%)	
Hospitalizations^a^, n	3,070	2,575	639	<0.001

Data were reported as n (%) for categorical variables.

*P*-value was computed using the Chi-squared test for categorical variables.

a Hospitalization, a hospital or intensive care unit.

Before the COVID-19 pandemic, the average number of patients visiting the ED for asthma per month was 673 (95% CI, 474–872). During the COVID-19 pandemic, from February to December 2020, there was an average of 176 patients per month (95% CI, 113–239), showing a decrease of 497 patients per month (P < 0.001), which was a decrease of 73.8%.

The rate of hospitalization after an ER visit was 38.25% (95% CI, 35.44–41.06%) on average per month before the COVID-19 pandemic and 32.46% (95% CI, 26.83–38.09%) during the COVID-19 pandemic, which was an average decrease of 5.79% (P = 0.004) (Table 2).

**Table 2:** Comparison of monthly ED visits and of asthma-related hospitalizations to ED visits by month between the pre-COVID-19 and COVID-19 pandemic periods.

Variable	Pre-COVID-19 (Feb. to Dec. 2018 and 2019)	COVID-19 pandemic (Feb. to Dec. 2020)	*P*-value
	N = 14,803	N = 1,932	
Monthly ED visits, n	673 ± 199	176 ± 63	<0.001
Age group			
2−6 years, n	335 ± 87	70 ± 34	<0.001
7−12 years, n	214 ± 74	60 ± 25	<0.001
13−18 years, n	125 ± 55	45 ± 10	<0.001
Ratio of asthma-elated hospitalizations to ED visits by month (%)	38.25 ± 2.81	32.46 ± 5.63	0.004

ED, emergency department; COVID-19, coronavirus disease.

**Fig. 3:**
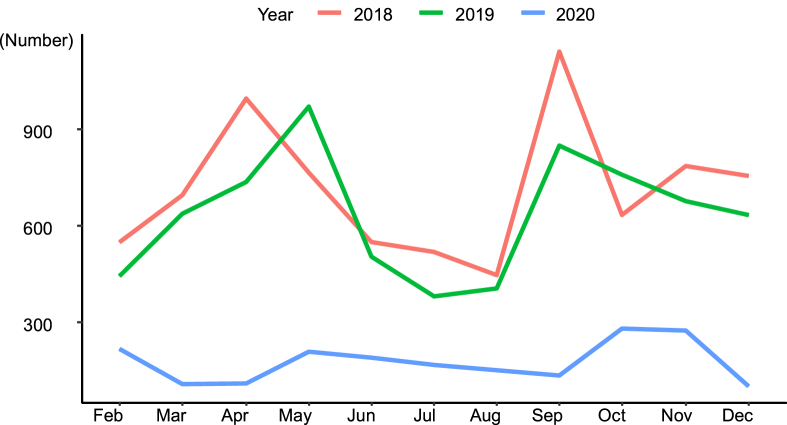
Monthly distribution of the number of emergency department visits for patients with asthma aged between 2 and 18 years from February to December in 2018, 2019, and 2020.

Asthma exacerbations showed sharp fluctuations in the number of patients depending on the season, peaking in spring and autumn. However, in 2020, during the COVID-19 pandemic, a different monthly pattern was observed. From February to December 2018, before the COVID-19 pandemic, the average monthly number of patients visiting the ER was 711 (95% CI, 593–830), compared to 994 and 1,140 in April and September, showing peaks in spring and autumn. The average monthly number of patients visiting the ED from February to December 2019 was 635 (95% CI, 528–741). In May, September, and October 2019, 969, 847, and 757 patients visited the ED, showing peaks in spring and autumn. From February to December 2020, during the period of the COVID-19 pandemic, the monthly average number of patients visiting the ED was 176 (95% CI, 112–239), and 280 and 272 patients in October and November, showing no peak during spring and only a peak during autumn (Fig. 3).

**Fig. 4:**
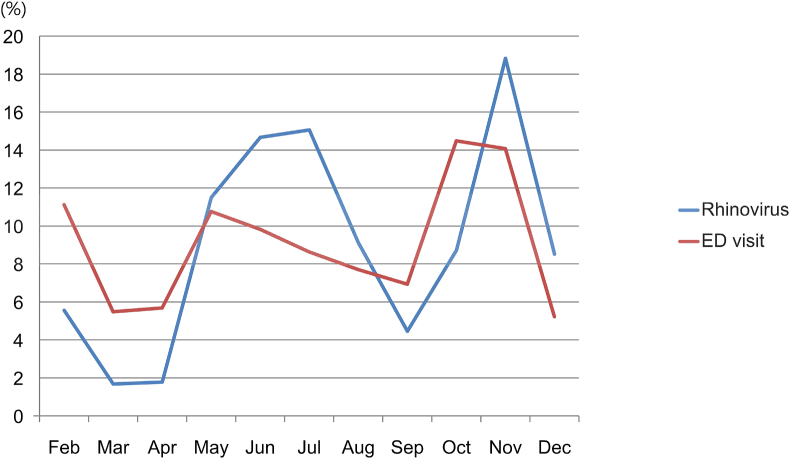
Comparison between the monthly ED visit ratio and monthly rhinovirus ratio during the coronavirus disease pandemic. ED, emergency department.

During the COVID-19 pandemic in 2020, the monthly distribution of patients visiting the ED with asthma exacerbations was compared with the monthly distribution of patients with rhinovirus to determine whether the asthma exacerbations were caused by a rhinovirus infection. The detection rate of rhinovirus was above the 95% CI in June, July, and November (95% CI, 38–123), and the number of patients visiting the ED with asthma exacerbations was higher than the 95% CI in October and November (95% CI, 112–239) (Fig. 4). ED visits due to asthma exacerbations and monthly detection patterns of rhinovirus did not coincide (Table 3).

**Table 3:** Comparison between the monthly ED visit ratio and monthly rhinovirus ratio during the coronavirus disease pandemic.

Variable	Rhinovirus (2020)	ED visits (2020)	X-squared	*P*-value
	100% (N = 1,009)	100% (N = 1,932)		
Month			153.46	<0.001
Feb	5.65% (57)	11.13% (215)		
Mar	1.68% (17)	5.49% (106)		
Apr	1.78% (18)	5.69% (110)		
May	11.50% (116)	10.77% (208)		
Jun	14.67% (148)	9.83% (190)		
Jul	15.06% (152)	8.64% (167)		
Aug	9.12% (92)	7.71% (149)		
Sep	4.46% (45)	6.94% (134)		
Oct	8.72% (88)	14.49% (280)		
Nov	18.83% (190)	14.08% (272)		
Dec	8.52% (86)	5.23% (101)		

*P*-value was computed by independent *t*-test for continuous variables.

ED, emergency department.

## Discussion

It is important to prevent exacerbations of asthma, a chronic disease, through continued management and prevention. The main exacerbating factor of asthma in children has been known to be a respiratory viral infection.^13^ If a respiratory viral infection was the cause of asthma exacerbations, it could be inferred that the frequency of asthma exacerbations in children and adolescents also would have decreased when the detection rate of respiratory viruses rapidly decreased during the COVID-19 pandemic in 2020, which was also confirmed in this study. During the COVID-19 pandemic, from February to December 2020, the detection rate of respiratory viruses decreased rapidly. Over the same period, the number of pediatric and adolescent patients visiting the ED with asthma exacerbations also decreased by 73.8% compared to that before the COVID-19 pandemic from February to December 2018 and from February to December 2019. A similar pattern can be confirmed in other countries over the same period. In a study of patients who visited the ED at Children's Hospital of Philadelphia with asthma exacerbations, the local government implemented stay-at-home and homeschooling policies in the week following the first confirmed case of COVID-19 on March 19, 2020. After that, the average number of patients visiting the ED due to asthma exacerbations was 5.8 per day, a decrease of 18.5 (76%) from the average of 24.3 per day in the previous 4 years.

While the COVID-19 pandemic continued, patients tended to avoid hospitals; therefore, it may be considered that the number of patients visiting the ED decreased as they did not visit the hospital despite having an asthma attack. A large-scale French study^14^ found that ED visits due to infectious diseases, such as colds, otitis media, and gastroenteritis, decreased during the COVID-19 pandemic, but visits due to non-infectious diseases, such as urinary tract infections, did not. Accordingly, there was no tendency for patients to avoid hospitals due to the COVID-19 pandemic. A multicenter study^15^ of 8 hospitals in the Netherlands compared the number of patients visiting the ED during the COVID-19 pandemic, from January to the end of June 2020, with that in the previous 4 years and reported that the hospitalization rate after visiting the ED remained constant despite a decrease in the number of patients visiting the ED with non-infectious diseases, suggesting that there was no tendency to avoid visiting the ED during the COVID-19 pandemic. If visits to the ED were delayed due to the tendency to avoid hospital visits, the rate of hospitalization would have increased as the severity of patients increased. However, no such phenomenon was observed, indicating that there was no avoidance of treatment. In this study, the proportion of patients hospitalized after ED treatment decreased during the COVID-19 pandemic. In contrast, the results of the current study showed that there might be no tendency to avoid visiting the ED as the proportion of patients hospitalized after ED treatment decreased, indicating no tendency of increased severity.

Air pollution indices checked as another independent variable in this study only decreased by 0.001 ppm in SO_2_ and 8 μg/m^3^ in PM10 during the COVID-19 pandemic, which could not explain the sharp decrease in the number of patients visiting the ER with asthma exacerbations. Therefore, the effect of air pollution on the exacerbation of asthma in children and adolescents seemed insignificant. A study^16^ on hospital visits by asthma patients in Philadelphia, USA, during the COVID-19 pandemic found that the number of patients with asthma exacerbations sharply decreased while the levels of 4 standard air pollutants (PM2.5, PM10, O_3_, and NO_2_) in Philadelphia did not change during the COVID-19 pandemic compared to that before the COVID-19 pandemic, suggesting that air pollution did not have a significant effect on asthma exacerbations.

Indoor inhaled allergens can be considered another exacerbating factor of asthma in children and adolescents during the COVID-19 pandemic. As children and adolescents spent more time at home due to social distancing and school closures, the time that they were exposed to indoor inhalant antigens, such as pets and house dust mites, increased. A study^17^ was conducted on indoor inhaled allergen sensitization before and after the COVID-19 pandemic, targeting children, adolescents, and adults with allergic diseases such as asthma, rhinitis, and atopic dermatitis at the Hospital of Guangzhou Medical University in China. The results indicated that the antigen-specific IgE values of 4 common indoor inhalant antigens, such as *Dermatophagoides pteronyssinus*, *Dermatophagoides farinae*, German cockroaches, and dog dander, significantly increased during the 2020 COVID-19 pandemic. Although the exposure to indoor inhalant allergens increased during this period, the number of patients with asthma exacerbations decreased, suggesting that the effect of indoor inhalant allergens on asthma exacerbations was insignificant.

Asthma is a disease with seasonal attacks,^18^ with the highest incidence in September in children and adolescents^19^ and a prevalence in spring.^20^ In this study, a peak was not observed during the COVID-19 pandemic in the spring of 2020, and patients with a 95% CI or higher visited the hospital in October and November 2020, showing a peak during autumn. According to a study^21^ by the Dana-Dwek Children's Hospital in Israel during the COVID-19 pandemic in 2020, the peak of patients with asthma exacerbations occurred in June 2020, which was 2 weeks after the end of the first of the most stringent and longest lockdowns in Israel (9 weeks from March 15, 2020, to May 17, 2020). In this study, the increase in patients with asthma at this time was interpreted as “back-to-school asthma,” which was caused by the easy transmission of respiratory viruses among children. However, in this study, the annual detection pattern of rhinovirus, which was almost exclusively detected during the COVID-19 pandemic, and the monthly distribution of patients with asthma exacerbations did not coincide. In our study, during the COVID-19 pandemic, the number of patients with a 95% CI or higher visited the hospital in October and November, showing an autumn peak. In particular, the number of patients with asthma exacerbations peaked in October, and the number of rhinovirus cases peaked in November. Since the peak of patients with asthma exacerbations preceded the peak of rhinovirus, this suggests that there were other exacerbating factors besides the rhinovirus infection. In addition to viral infection, it is possible to consider other causes, such as changes in temperature, atmospheric pressure, or pollen concentration.

By comparing the monthly patterns of pediatric asthma exacerbation cases and major factors of pediatric asthma exacerbation for 2020 when respiratory viral infection, a major factor of pediatric asthma exacerbation was eliminated, the cause of the fall peak of pediatric asthma exacerbation could be inferred. For example, South Korea has large fluctuations in temperature and air pressure during fall. Assuming that fluctuations in temperature and air pressure partially contribute to asthma exacerbation during fall, this can be confirmed by comparing the monthly patterns of asthma exacerbation cases and fluctuations in temperature and air pressure in 2020. The same method can be used for comparison with monthly pattern of pollens as well.

A study reporting changes in temperature and air pressure as one of the causes of asthma exacerbations in children and adolescents in the autumn have shown that asthma symptoms exacerbate when the temperature is low or the temperature drops rapidly.^22^ Hashimoto et al^23^ reported that ED visits increased when air pressure, temperature, or humidity suddenly dropped after being high, and asthma exacerbations were common in spring and autumn due to such unstable atmospheric changes.

A seasonal increase in pollen concentration has also been studied as a causative factor for asthma exacerbations. However, a recent domestic study argued that it was difficult to see a distinct increase in pollen concentration for each season as the cause of asthma exacerbations in the autumn.^24^ A study^25^ that analyzed patients who visited the ED at Children's Hospital of Philadelphia during the 2020 COVID-19 pandemic reported a sharp drop in asthma exacerbations despite the emergence of COVID-19 in the Northeast US during spring when pollen concentrations were high, suggesting that pollen levels did not significantly affect asthma exacerbations.

This study presented representative large-scale data on pediatric and adolescent patients with asthma who visited EDs nationwide for 3 years in South Korea and investigated the incidence of asthma exacerbations due to respiratory viral infections based on a sharp decrease in respiratory viral infections due to social distancing during the COVID-19 pandemic. Prevention of respiratory viral infections was the most effective way to prevent asthma exacerbations. However, the possibility of asthma exacerbation due to factors other than respiratory viral infection was also identified by confirming that the prevalence distribution of rhinovirus during the COVID-19 pandemic did not coincide with the distribution of monthly hospital visits in patients with asthma exacerbations.

The limitation of this study was that the study participants were patients who visited the ED, and did not include outpatients; therefore, it could not be representative of all patients with asthma. Patients who visit the ED tend to have a rapid exacerbation of symptoms compared to outpatients. Therefore, since patients whose main cause of rapid exacerbation of symptoms was a viral infection were selected as the participants of the study, viral infection may have been overinterpreted as a cause of exacerbation of asthma. In addition, as the NEDIS data used in this study did not include the clinical features and test results of patients admitted to the ED, it was not possible to analyze the effect of individual patient characteristics on asthma exacerbation.

## Conclusion

Respiratory viral infections are associated with asthma exacerbations in children and adolescents. In this study, air pollution is not a major factor for ED visits due to asthma exacerbations. Even though the prevalence of respiratory viruses is decreasing, emergency room visits due to worsening asthma are trending in the fall. This phenomenon may indicate that asthma has worsened due to other causes such as pollen or fluctuations in temperature and air pressure.

## Abbreviations

ED, emergency department; COVID-19, coronavirus disease 2019; NEDIS, National Emergency Department Information System; SO_2_, sulfur dioxide; NO_2_, nitrogen dioxide; O_3_, ozone; CO, carbon monoxide; PM, particulate matter; CI, confidence interval.

## Funding

This work was supported by research fund of Chungnam National University.

## Availability of data and materials

The datasets used and/or analyzed during the current study are available from the corresponding author on reasonable request.

## Author contributions

Conceptualization: YKW, SIC, and EHC; Methodology and analysis: YKW, SIC, and EHC; Interpretation of data: YKW, SIC, and EHC; Writing the manuscript: YKW.

## Ethics statement

The study was approved by the Ethics Committee of the Chungnam National University School of Medicine in 2021 (ID number CNUH 2021-06-056).

## Consent for publication

The authors have read and approved the final version of the manuscript, its content, and its submission to *World Allergy Organization* *Journal*. We confirm that this manuscript is original, has not been published before, is not currently being considered for publication elsewhere, and has not been posted to a preprint server.

## Declaration of competing interest

The authors report no competing interests.

## References

[1] Busse W.W., Lemanske R.F. (2001). Asthma. N Engl J Med.

[2] Johnston S.L., Pattemore P.K., Sanderson G. (1995). Community study of role of viral infections in exacerbations of asthma in 9-11 year old children. BMJ.

[3] Won Y.K., Hwang T.H., Roh E.J., Chung E.H. (2016). Seasonal patterns of asthma in children and adolescents presenting at emergency departments in Korea. Allergy Asthma Immunol Res.

[4] Perez L., Declercq C., Iñiguez C. (2013). Chronic burden of near-roadway traffic pollution in 10 European cities (APHEKOM network). Eur Respir J.

[5] Park I.N., Yum H.K. (2020). Stepwise strategy of social distancing in Korea. J Kor Med Sci.

[6] Korea Ministry of Health and Welfare. COVID-19. Sejong (Korea): Ministry of Health and Welfare. https://ncov.kdca.go.kr. Accessed 15 August 2022.

[7] Huh K., Jung J., Hong J. (2021). Impact of nonpharmaceutical interventions on the incidence of respiratory infections during the coronavirus disease 2019 (COVID-19) outbreak in Korea: a nationwide surveillance study. Clin Infect Dis.

[8] Wang M., Liu F., Zheng M. (2021). Air quality improvement from COVID-19 lockdown: evidence from China. Air Qual Atmos Health.

[9] Infectious Disease Homepage. Korea Influenza & Respiratory Viruses Surveillance System (KINRESS) data. Cheongju (Korea): Korea Disease Control and Prevention Agency. https://www.kdca.go.kr/npt/biz/npp/portal/nppPblctDtaMain.do?pblctDtaSeAt=3. Accessed 15 August 2022.

[10] Cha Jeongok, Seo Yejin, Kang Seulki, Kim Inho, Gwack∗ Jin (2023). Sentinel surveillance results for influenza and acute respiratory infections during the coronavirus disease 2019 pandemic. Public Health Weekly Report.

[11] Lee Nam-Joo, Woo SangHee, Lee Jaehee, Rhee Jee Eun, Kim∗ Eun-Jin (2023). 2021-2022 influenza and respiratory viruses laboratory surveillance report in the Republic of Korea. Public Health Weekly Report.

[12] Air Korea. Air pollution data. Incheon (Korea): Korea Environment Corporation.https://www.airkorea.or.kr/web/detailViewDown?pMENU_NO=125. 15 August 2022.

[13] Busse W.W., Lemanske RF Jr, Gern J.E. (2010). Role of viral respiratory infections in asthma and asthma exacerbations. Lancet.

[14] Angoulvant F., Ouldali N., Yang D.D. (2021). Coronavirus disease 2019 pandemic: impact caused by school closure and national lockdown on pediatric visits and admissions for viral and nonviral infections- a time series analysis. Clin Infect Dis.

[15] Kruizinga M.D., Peeters D., van Veen M. (2021). The impact of lockdown on pediatric ED visits and hospital admissions during the COVID19 pandemic: a multicenter analysis and review of the literature. Eur J Pediatr.

[16] Taquechel K., Diwadkar A.R., Sayed S. (2020). Pediatric asthma health care utilization, viral testing, and air pollution changes during the COVID-19 pandemic. J Allergy Clin Immunol Pract.

[17] Li Y., Hu H., Zhang T. (2021). Increase in indoor inhalant allergen sensitivity during the COVID-19 pandemic in South China: a cross-sectional study from 2017 to 2020. J Asthma Allergy.

[18] Global Initiative for Asthma (2022). Global strategy for asthma management and prevention (2022 update). https://ginasthma.org/wp-content/uploads/2022/07/GINA-Main-Report-2022-FINAL-22-07-01-WMS.pdf.

[19] Cohen H.A., Blau H., Hoshen M., Batat E., Balicer R.D. (2014). Seasonality of asthma: a retrospective population study. Pediatrics.

[20] Wisniewski J.A., McLaughlin A.P., Stenger P.J. (2016). A comparison of seasonal trends in asthma exacerbations among children from geographic regions with different climates. Allergy Asthma Proc.

[21] Be'er M., Amirav I., Cahal M. (2022). Unforeseen changes in seasonality of pediatric respiratory illnesses during the first COVID-19 pandemic year. Pediatr Pulmonol.

[22] Celenza A., Fothergill J., Kupek E., Shaw R.J. (1996). Thunderstorm associated asthma: a detailed analysis of environmental factors. BMJ.

[23] Hashimoto M., Fukuda T., Shimizu T. (2004). Influence of climate factors on emergency visits for childhood asthma attack. Pediatr Int.

[24] Oh J.W., Lee H.B., Kang I.J. (2012). The revised edition of Korean calendar for allergenic pollens. Allergy Asthma Immunol Res.

[25] Kenyon C.C., Hill D.A., Henrickson S.E., Bryant-Stephens T.C., Zorc J.J. (2020). Initial effects of the COVID-19 pandemic on pediatric asthma emergency department utilization. J Allergy Clin Immunol Pract.

